# Antioxidant property and characterization data of 1-*o*-galloylglycerol synthesized via enzymatic glycerolysis

**DOI:** 10.1016/j.dib.2020.105110

**Published:** 2020-01-14

**Authors:** Siyu Zhang, Casimir C. Akoh

**Affiliations:** Department of Food Science & Technology, University of Georgia, Athens, GA, 30602, USA

**Keywords:** 1-*o*-Galloylglycerol, Gallic acid derivatives, Lipase-catalyzed glycerolysis, Spectroscopic, Antioxidant activity

## Abstract

This article provides comprehensive experimental data characterizing antioxidant activity, as well as chemical and physical properties of 1-*o*-galloylglycerol (GG), synthesized by enzymatic glycerolysis of propyl gallate (PG) using a food-grade lipase (Lipozyme® 435) [1]. GG was characterized by Fourier-transform infrared spectroscopy (FT-IR), ^1^H, ^13^C, ^1^H–^1^H gradient correlation spectroscopy (gCOSY), ^1^H–^13^C gradient heteronuclear single quantum coherence (gHSQC), ^1^H–^13^C gradient heteronuclear multiple quantum coherence (gHMQC), and ^1^H–^13^C gradient heteronuclear multiple bond correlation (gHMBC) nuclear magnetic resonance spectroscopies (NMR), and ultraviolet–visible spectrophotometry (UV–Vis). The antioxidant property of GG, which was evaluated through 1,1-diphenyl-2-picrylhydrazyl (DPPH^•^), 2,2′-azinobis (3-ethylbenzthiazoline-6-sulfonic acid) (ABTS^•+^), ferric reducing antioxidant power (FRAP), and hydrogen peroxide (H_2_O_2_) scavenging assays, is also presented.

**Table:** Specifications Table

Subject	Food Science
Specific subject area	Antioxidant
Type of data	Table, Figure, FT-IR spectra, NMR spectra, UV–Vis spectra
How data were acquired	NMR: Varian Unity Inova 500 MHz NMR Spectrometer equipped with a 5 mm hydrogen-carbon-nitrogen room temperature probe. NMR spectra were analyzed with MestreNova NMR software. FT-IR: Nicolet Nexus FT-IR 1100 spectrometer equipped with a ZnSe attenuated total reflection attachment. The data were processed using Omnilab software from Omnilab Group and KnowItAll® informatic system from Bio-Rad Laboratories. UV–Vis spectrophotometry: Shimadzu UV-1601 UV–Vis spectrophotometer.
Data format	Raw (within the data article as a supplementary file), Analyzed
Parameters for data collection	All data were collected from experiments described in methods section conducted in triplicate.
Description of data collection	All data were collected from experiments described in methods section conducted in triplicate.
Data source location	University of Georgia, Athens, GA, US, 30602
Data accessibility	With the article
Related research article	Zhang, S., & Akoh, C. C. (2020). Enzymatic synthesis of 1-*o*-galloylglycerol: Characterization and determination of its antioxidant properties. *Food Chemistry, 305*, 125479. https://doi.org/10.1016/j.foodchem.2019.125479.

**Table:** 

**Value of the Data** • The described method is facile and efficient, providing an enzymatic method for obtaining 1-*o*-galloylglycerol, a water-soluble gallic acid derivative. • 1-*o*-Galloylglycerol was characterized by NMR, FT-IR and UV–Vis spectrophotometry. The spectroscopic data information can be manipulated for advanced molecular studies of biological active molecules. • The antioxidant property of 1-*o*-galloylglycerol was obtained by various *in vitro* antioxidant assays, demonstrating its potential for use in aqueous-based food, cosmetic, and pharmaceutical products. • Detailed methods and calculations of four common *in vitro* antioxidant assays are provided in this article. Readers who are interested in using these assays could easily follow the provided instructions.

## 1 Data description

**Fig. 1:**
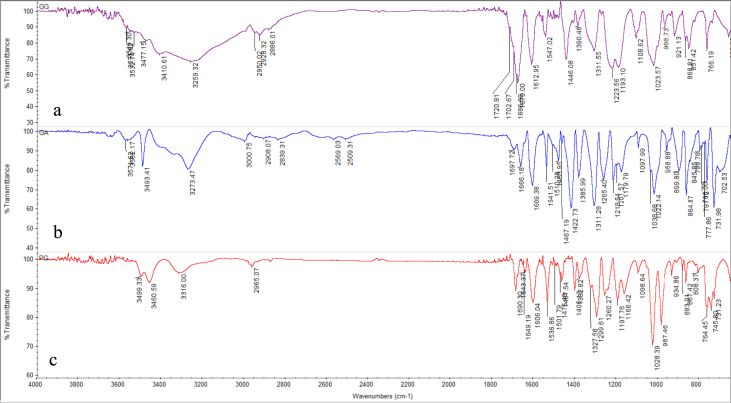
FT-IR spectra of GG (a), GA (b), and PG(c). GG, 1-o-galloylglycerol; GA, gallic acid; PG, propyl gallate.

**Fig. 2:**
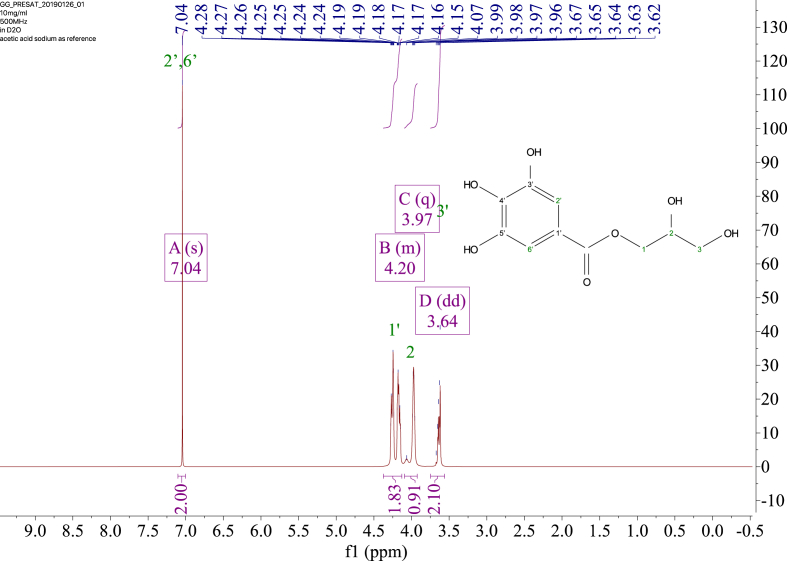
^1^H NMR spectrum of 1-o-galloylglycerol (GG).

**Fig. 3:**
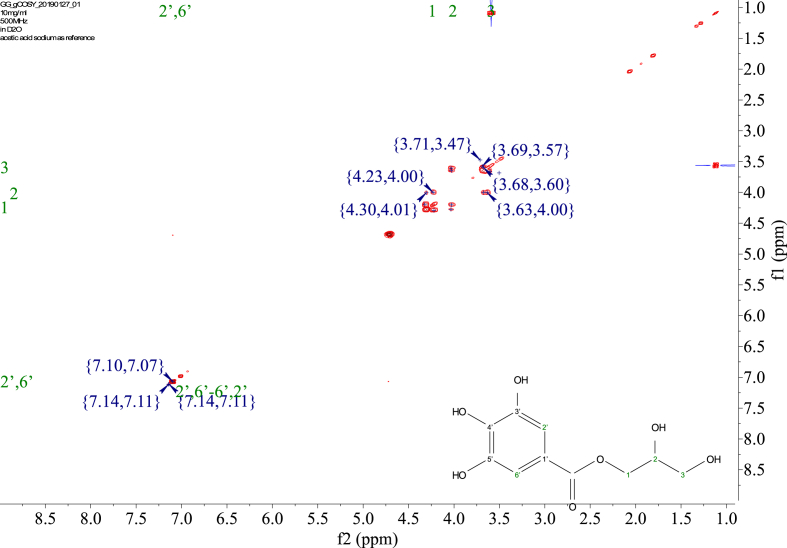
gCOSY spectrum of 1-o-galloylglycerol (GG).

**Fig. 4:**
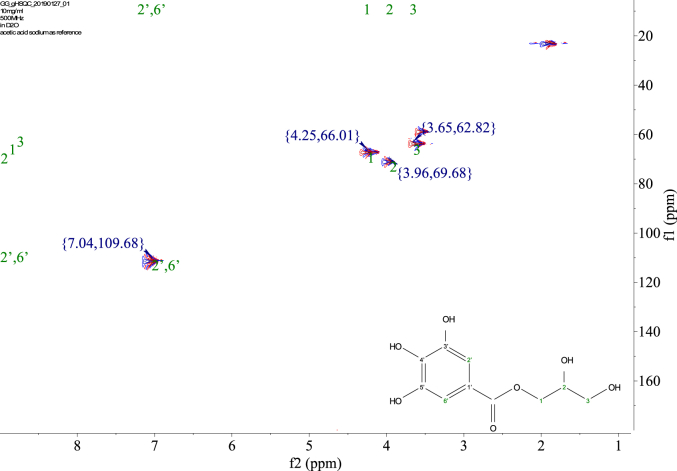
gHSQC spectrum of 1-o-galloylglycerol (GG).

**Fig. 5:**
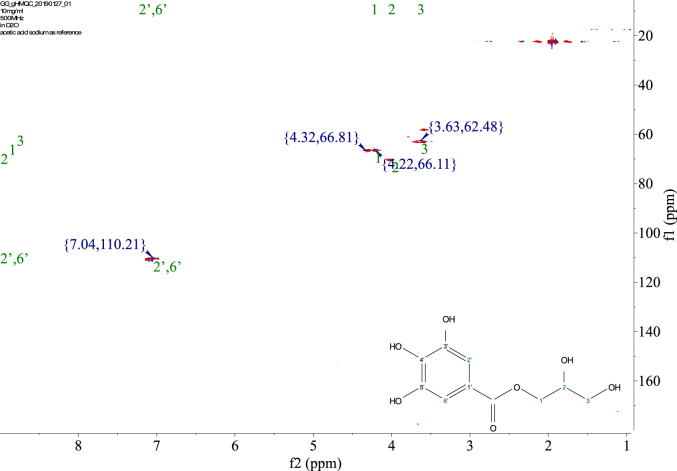
gHMQC spectrum of 1-o-galloylglycerol (GG).

**Fig. 6:**
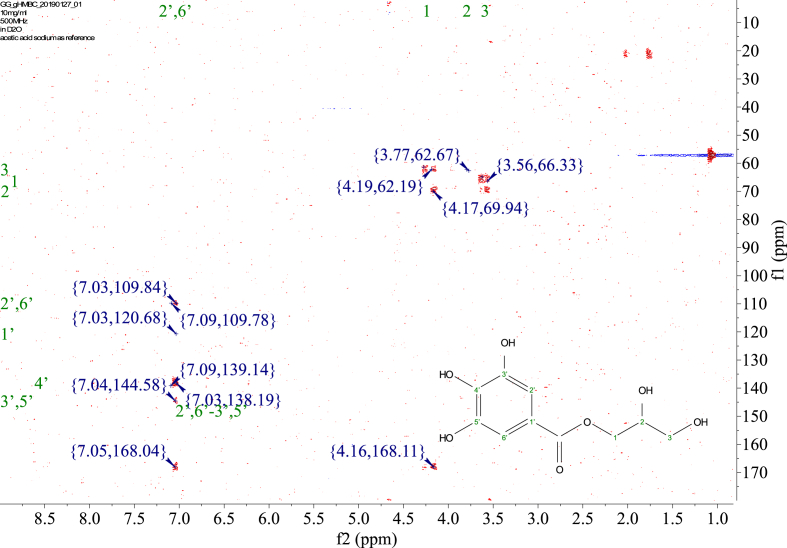
gHMBC spectrum of 1-o-galloylglycerol (GG).

**Fig. 7:**
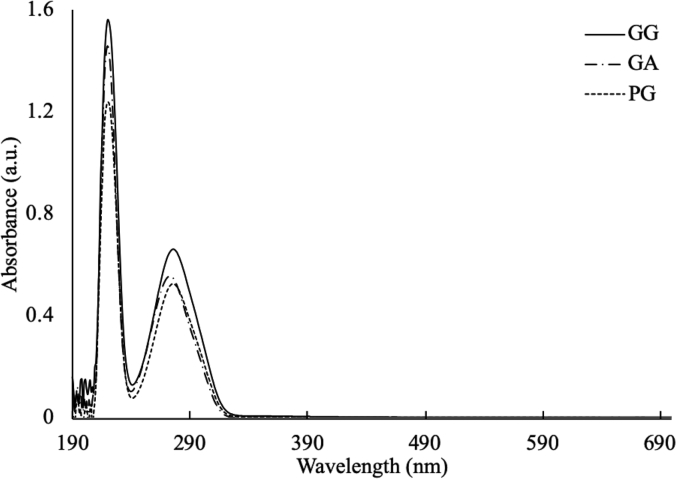
UV–Vis spectra of 1-o-galloylglycerol (GG), gallic acid (GA), and propyl gallate (PG).

**Fig. 8:**
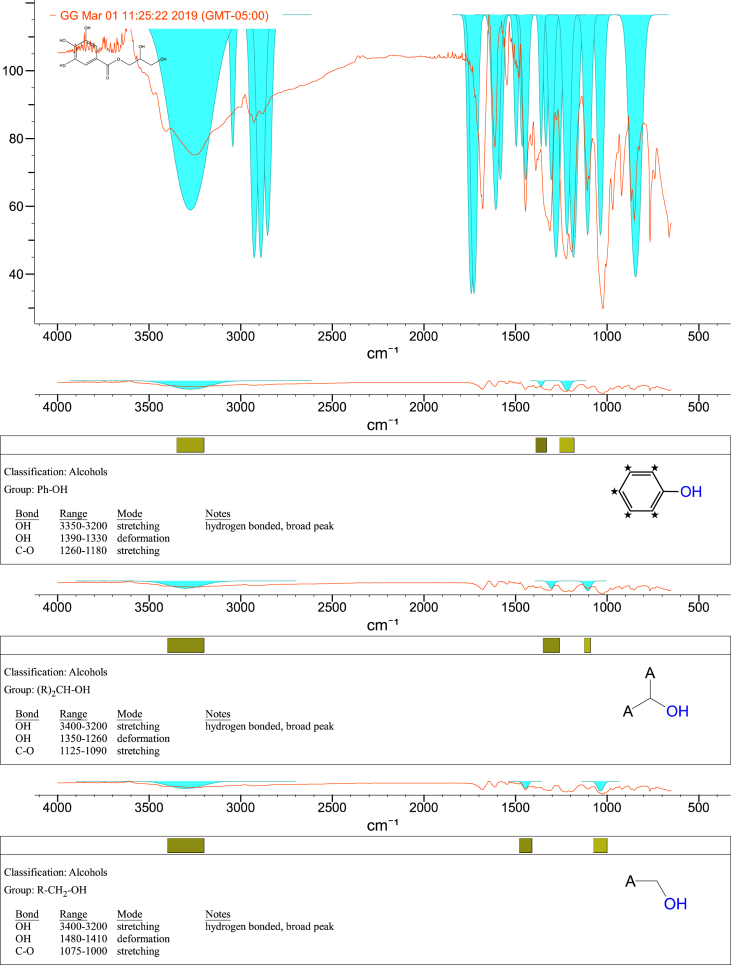

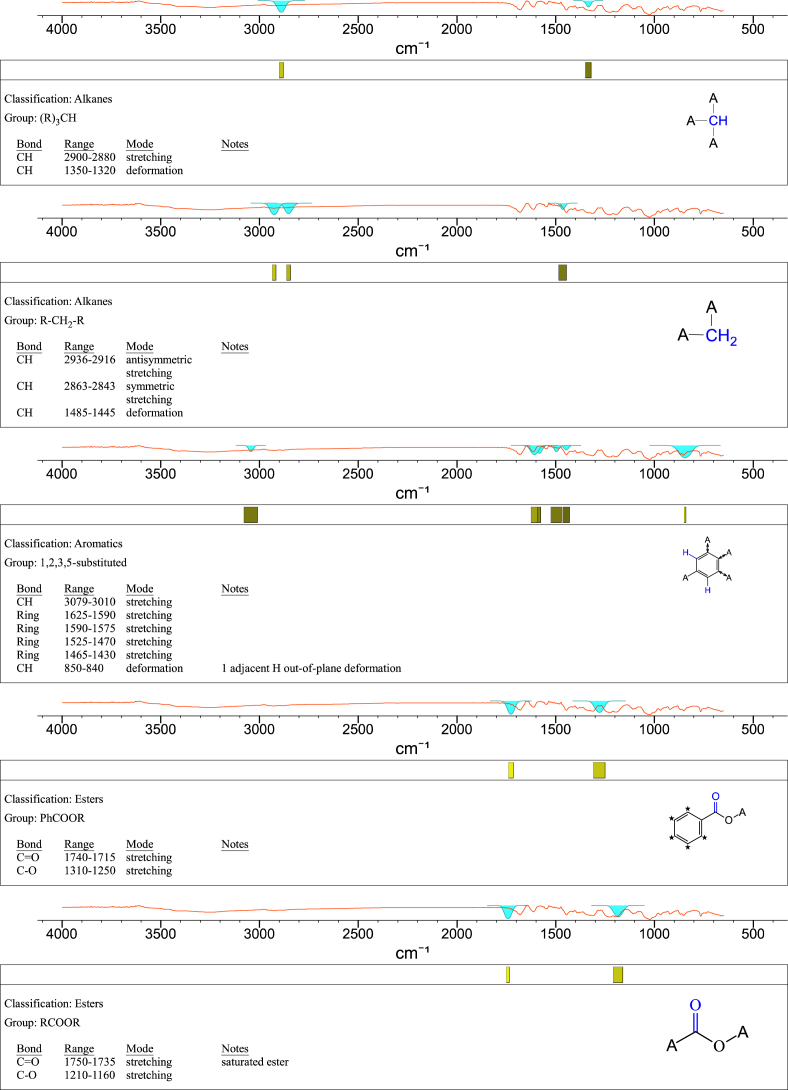
IR spectrum interpretation report of 1-*o*-galloylglycerol (GG).

**Fig. 9:**
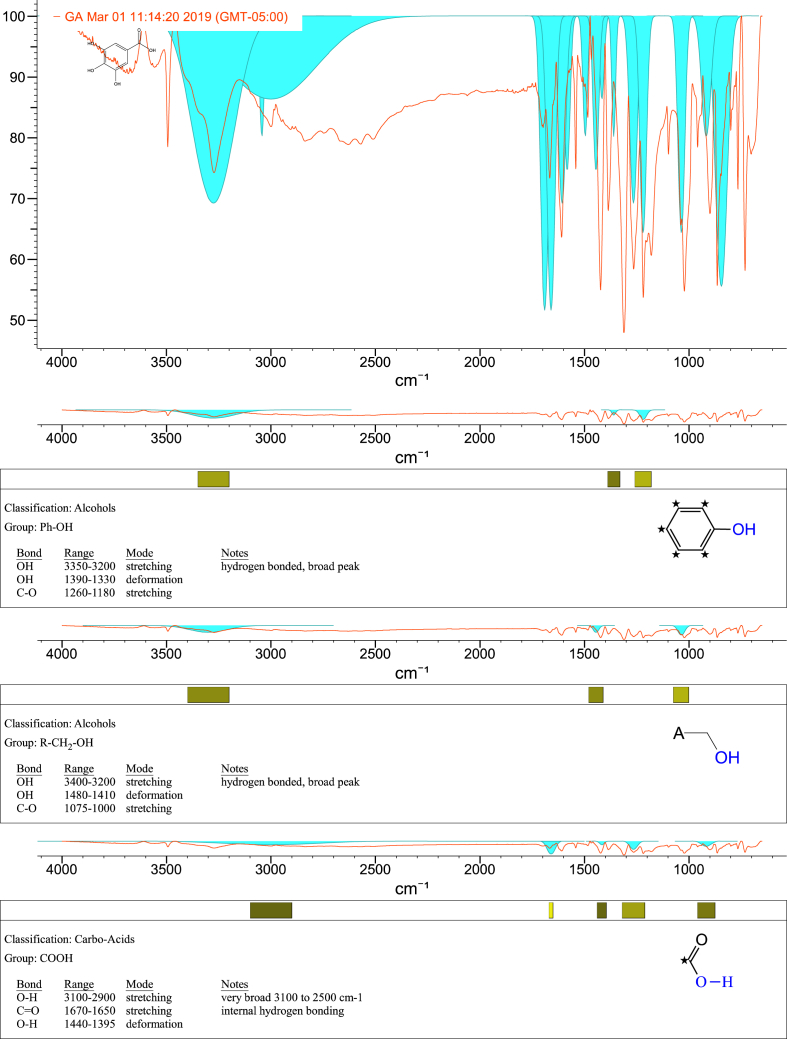

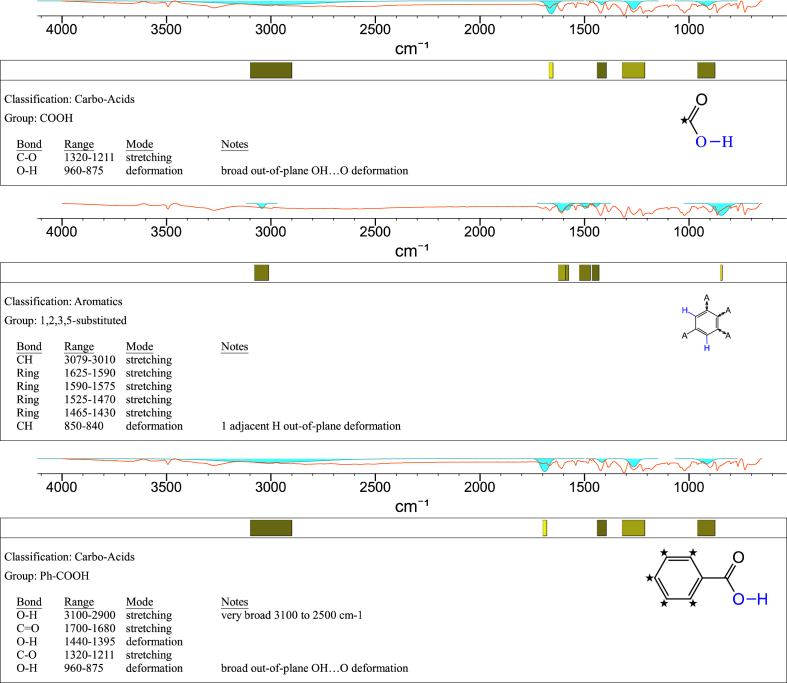
IR spectrum interpretation report of gallic acid (GA).

**Fig. 10:**
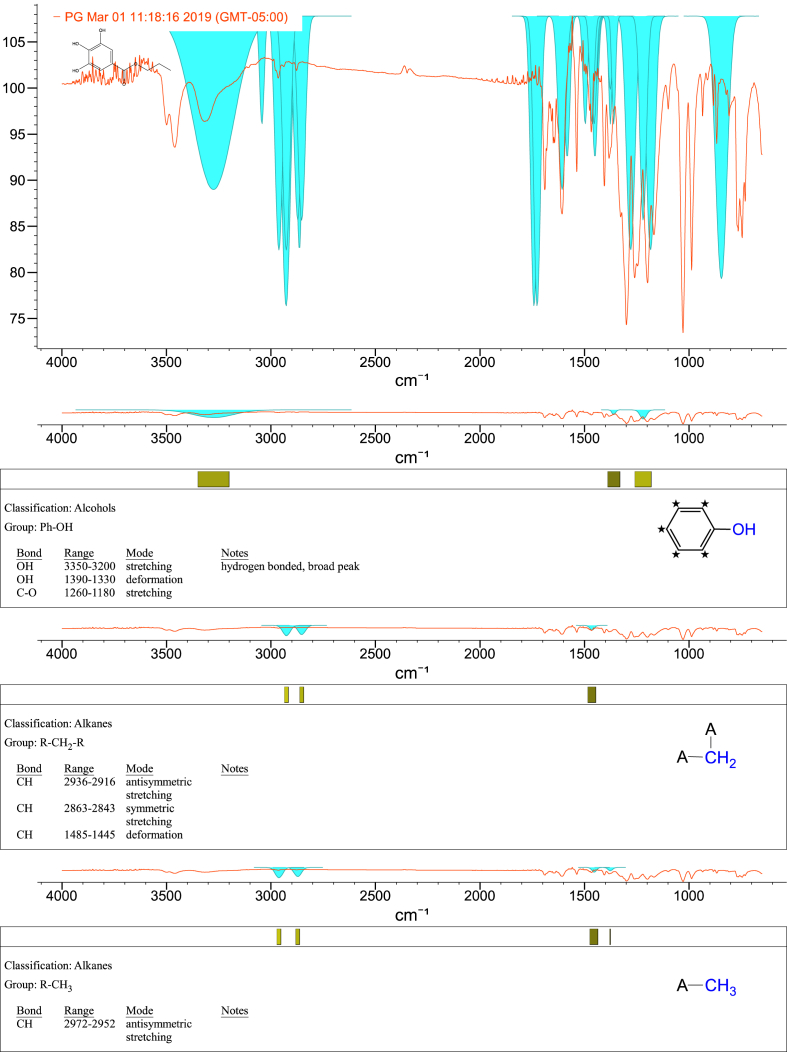

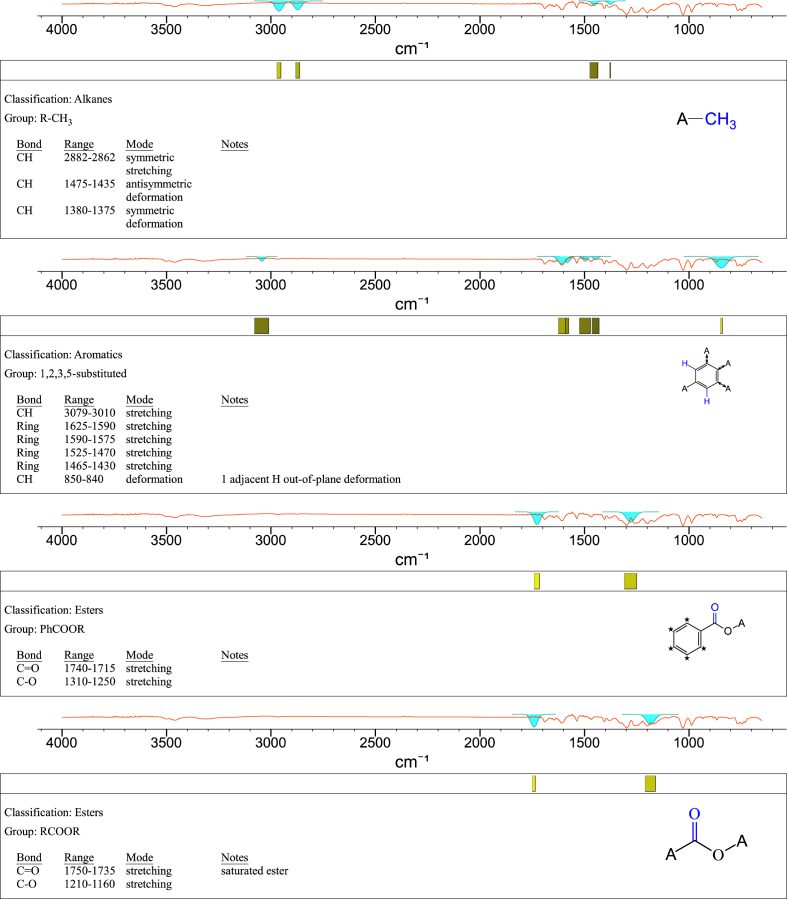
IR spectrum interpretation report of propyl gallate (PG).

1-*o*-Galloylglycerol (GG) was synthesized by the enzymatic glycerolysis of propyl gallate (PG) using a food-grade lipase (Lipozyme® 435). This article provides comprehensive experimental data characterizing antioxidant activity, as well as chemical and physical properties of GG, given in Ref. [1]. The reaction parameters, such as reaction time (120 h), reaction temperature (50 °C), substrate ratio (glycerol/PG = 25/1 mol/mol), and enzyme load (23.8% w/w), were chosen based on previous report [2]. After separation and purification from the reaction mixture, GG was characterized by Fourier-transform infrared spectroscopy (FT-IR) (Fig. 1), ^1^H (Fig. 2), ^1^H–^1^H gradient correlation spectroscopy (gCOSY) (Fig. 3), ^1^H–^13^C gradient heteronuclear single quantum coherence (gHSQC) (Fig. 4), ^1^H–^13^C gradient heteronuclear multiple quantum coherence (gHMQC) (Fig. 5), and ^1^H–^13^C gradient heteronuclear multiple bond correlation (gHMBC) (Fig. 6) nuclear magnetic resonance (NMR) and UV–Visible spectrophotometry (Fig. 7). The raw spectral files of FT-IR are presented in supplementary information as spa files. UV–Vis spectra of GG, gallic acid (GA), and PG are also provided as csv files in the supplementary materials. The NMR spectral files built in pdf format are provided in supplementary materials, as well as their acquisition parameters. Functional groups and their interaction of GG, GA, and PG were analyzed and compared using FT-IR spectral data. The interpretations of FT-IR spectra are shown in Fig. 8, Fig. 9, and Fig. 10 using KnowItAll® informatic system (Bio-Rad Laboratories, Hercules, CA, USA). The spectrophotometric data of DPPH^•^, ABTS^•+^, FRAP, and H_2_O_2_ *in vitro* antioxidant assays are shown in supplementary materials in xlsx format. Details of the standard curves used in these assays are also provided in Table 1. The DPPH^•^ scavenging kinetics of GG, GA, and PG at the concentrations of 20, 10, 5, and 2 μM were tested in methanol and are also presented in supplementary materials.

**Table 1:** Calibration curves used in ABTS^•+^, FRAP, and H_2_O_2_ assays.

Assay and calibration curve	ABTS^•+^	FRAP	H_2_O_2_	
	Trolox^a^	FeSO_4_^b^	H_2_O_2_^c^	Trolox^d^
Coefficient of determination	0.994	0.999	0.999	0.996
Tested concentration	10, 20, 50, 100, 200 μM	100, 200, 500, 1000, 2000 μM	0, 2, 5, 10, 20 ppm	20, 40, 200, 400 μM

a Expressed as concentration of Trolox ethanol solution against percentage of scavenged ABTS^•+^.

b Expressed as concentration of FeSO_4_ aqueous solution against absorbance.

c Expressed as concentration of H_2_O_2_ aqueous solution against absorbance.

d Expressed as concentration of Trolox methanol solution against percentage of scavenged H_2_O_2_.

## 2 Experimental design, materials, and methods

The enzymatic glycerolysis of PG was performed in a 100-mL double-layer jacketed glass reactor equipped with a circulating water bath, stirred with a PTFE anchor paddle stirring rod, and using glycerol as both the reactant and solvent. The purified GG was structurally characterized by UV–Vis, FT-IR and NMR spectroscopies.

A Nicolet Nexus FT-IR 1100 spectrometer (Thermo Fisher Scientific Co. Ltd., Waltham, MA, USA) equipped with a ZnSe attenuated total reflection attachment was used to collect the FT-IR spectra of samples (νmax was reported in cm^−1^). Before each experiment, the instrument was purged with nitrogen for at least 10 min. Then, 50 mg of the samples were directly placed onto the ZnSe crystal and pressed using the attached accessory. The spectra were collected from 650 to 4000 cm^−1^ with a resolution of 4 cm^−1^ and 32 scans. The data were processed using Omnilab software (Omnilab Group, Bremen, Germany) and the KnowItAll® informatic system (Bio-Rad Laboratories, Hercules, CA, USA).

NMR spectroscopy analysis was done with 10 mg of the sample dissolved in D_2_O with 10 mM acetic-2-^13^C acid sodium salt as the internal standard. ^1^H, gCOSY, gHSQC, gHMQC, and gHMBC spectra were recorded at 25 °C using a Varian Unity Inova 500 MHz NMR Spectrometer (Varian Inc., Palo Alto, CA, USA) equipped with an 8-mm hydrogen-carbon-nitrogen room temperature probe. The chemical shifts of GG were reported in parts per million (δ/ppm). Acetic-2-^13^C acid sodium salt (δH/δC 1.90/25.63, 164.15 ppm) was used as the internal standard. The chemical shifts were assigned based on the 1D and 2D NMR spectra (Fig. 2, Fig. 3, Fig. 4, Fig. 5, Fig. 6). In ^1^H spectrum (Fig. 2), only peaks of GG are presented, signals from solvent residuals were suppressed using presaturation technique with default parameters of the NMR instrument. No signal suppression was used for gCOSY (Fig. 3), gHSQC (Fig. 4), gHMQC (Fig. 5), and gHMBC (Fig. 6) NMR spectroscopies. Signals from solvent residuals (water and ethanol) and internal standard (acetic-2-^13^C acid sodium salt) are shown in 2D spectra (Fig. 3, Fig. 4, Fig. 5, Fig. 6) along with the signals of GG. The acquired raw spectral data were processed with MestreNova NMR software (Santiago de Compostela, Spain). All the acquisition parameters are provided in supplementary information.

The UV–Vis spectra of the samples were measured using a UV-1601 UV–Vis spectrophotometer (Shimadzu, Kyoto, Japan). The samples were dissolved in methanol at a concentration of 50 μM, then scanned over the range from 190 to 700 nm with a 0.5 nm scan interval and a medium scan speed.

The DPPH^•^ scavenging assay was performed according to a previous study [3] with some modifications. Samples of GG, PG, and GA were dissolved in methanol at different concentrations (2, 5, 10, and 20 μM). DPPH was dissolved in methanol to make a solution at a concentration of 200 μM. Equal amounts of DPPH^•^ and sample solutions were mixed and then monitored spectrophotometrically at 517 nm at a 1 s interval for 30 min using the UV-1601UV–Vis spectrophotometer mentioned previously. Instead of antioxidant solution, ultrapure water was used in the control groups. The results were expressed as the remaining percentage of DPPH^•^ after being reduced by the samples. All experiments were performed in triplicate. All the samples and reagents were freshly prepared daily.

An improved ABTS radical cation decolorization assay [4] with modifications [5] was used. Briefly, ABTS^•+^ was produced by reacting 7 mM ABTS water solution with 2.45 mM potassium persulfate in the dark at room temperature for 16 h. The ABTS^•+^ solution was then diluted with ethanol to obtain an absorbance of 0.70 (±0.01) at a wavelength of 734 nm. Samples of GG, PG, and GG were dissolved in ethanol to obtain solutions at concentrations of 10, 20, 40, 50, and 100 μM. Trolox™ was used as the standard for measuring the antioxidant activity of the samples. Ethanol solutions of Trolox™ were prepared at concentrations of 10, 20, 50, 100, and 200 μM. Ethanol was used for the control groups. A 100-μL sample was mixed with 900 μL of the ABTS^•+^ solution, then the mixture was incubated in the dark at 30 °C for 6 min. The absorbance of the mixture was measured with the spectrophotometer mentioned previously at 734 nm. The results were expressed as the decrease in absorbance after mixing the samples, compared with the control groups. All experiments were performed in triplicate. All the samples and reagents were freshly prepared daily.

The FRAP assay was conducted in accordance with previous studies [6,7] with modifications. The FRAP reagent was prepared by mixing solutions of 10 mM TPTZ (in 40 mM HCl), 20 mM FeCl_3_, and 300 mM acetate buffer (pH 3.6) at a volumetric ratio of 1:1:10. Aqueous solutions of FeSO_4_•7H_2_O were prepared at concentrations of 100, 200, 500, and 1000 μM, to be used for calibration. Antioxidant samples of GG, PG, and GA were also dissolved in water at various concentrations (20, 40, 100, 200, and 400 μM). Water was used as the blank. Freshly prepared FRAP reagent (900 μL) was mixed with 30 μL of the sample solution, then with 90 μL water. Thus, the final dilution of the sample in the reaction mixture was 34 times. The reaction mixture was then incubated in the dark at 37 °C for 4 min. The absorbance of the reaction mixture was monitored at 593 nm. The increase in absorbance was checked with the calibration curve of FeSO_4_•7H_2_O solution to give the results expressed as Fe^2+^ equivalents.

The hydroxyl radical scavenging ability of the compounds was measured using the H_2_O_2_ scavenging assay [8,9] with modifications. Solutions of GG, PG, and GA in methanol were prepared at various concentrations (20, 40, 200, and 400 μM). Equal amounts of the sample solution and 0.002% (w/w) H_2_O_2_ solution were mixed with 0.8 mL PBS and incubated in the dark at 37 °C for 10 min. One milliliter assay reagent, containing 0.2 mg/mL phenol red and 0.1 mg/mL horseradish peroxidase in PBS, was added and incubated under the same conditions for 15 min. After incubation, 50 μL of 1 M sodium hydroxide solution were added and the absorbance of the mixture was measured immediately at 610 nm using spectrophotometer. H_2_O_2_ solutions at different concentrations (0.0002, 0.0005, 0.001, and 0.002%, w/w) were used as the calibration curve. The decrease in absorbance (compared with 0.002% w/w H_2_O_2_ solution) was expressed against the calibration curve of the H_2_O_2_ solutions. The results were expressed as the percentages of scavenged H_2_O_2_.

The absorbances of GG, GA, and PG of four *in vitro* antioxidant assays are provided in supplementary materials. The results of all four antioxidant assays were calculated using regression analysis and ANOVA by JMP® software (version 13.2.0, SAS Institute, Inc., Cary, NC, USA), and presented as means followed by standard errors.
