# Supplementary figures and images for: Antioxidant property and characterization data of 1-o-galloylglycerol synthesized via enzymatic glycerolysis
Source: Data Brief. 2020 Jan 14;29:105110. doi: 10.1016/j.dib.2020.105110 (PMC6974738; doi:10.1016/j.dib.2020.105110)

# Stacked Spectrum Graph Report

03/04/2019 02:54:11 PM

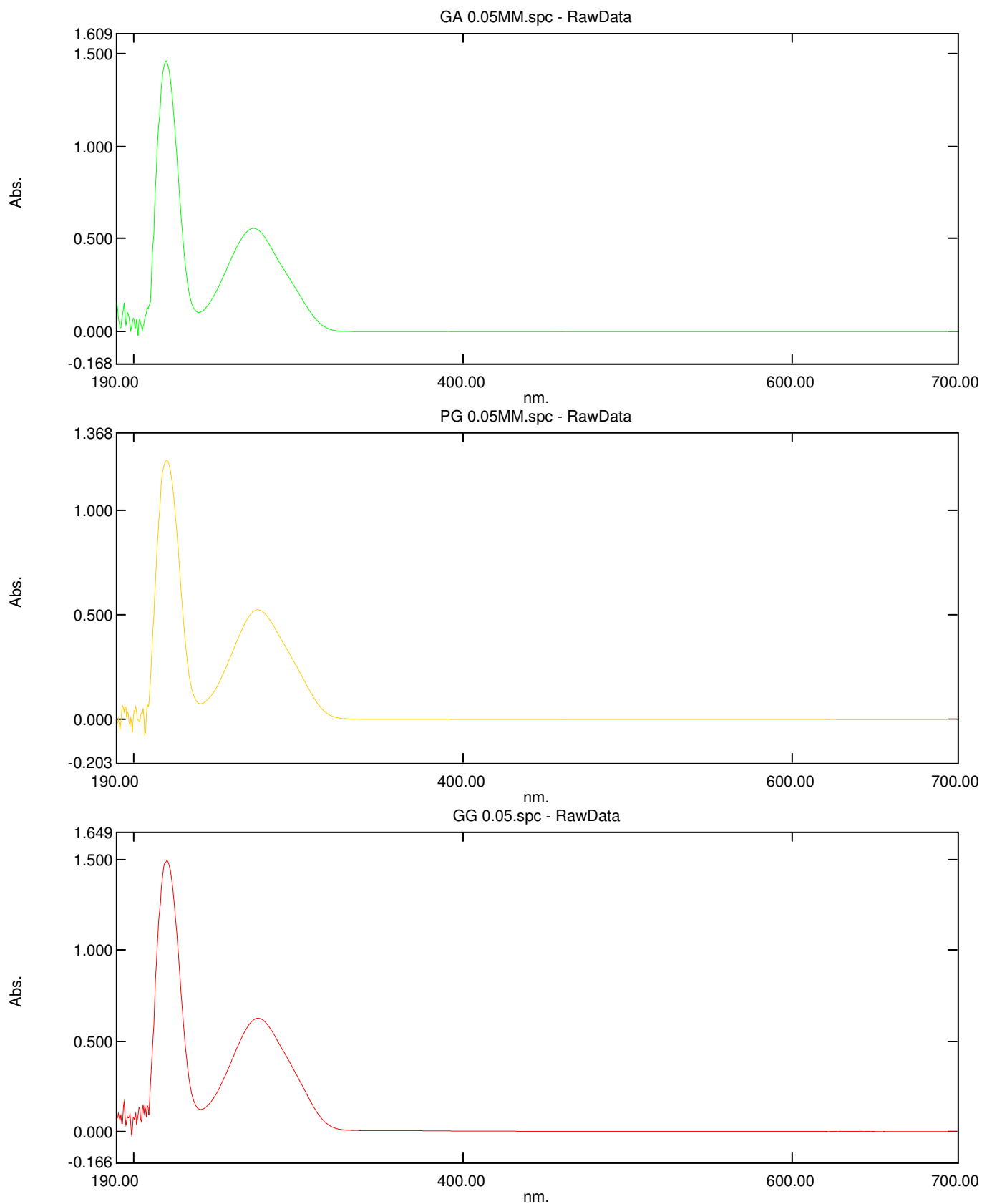

Supplement: Multimedia component 1 [file mmc1.zip › Supplementary materials/UV/Spc Stacked Graph.pdf]

GG\_PRESAT\_20190126\_01  
10mg/ml  
500MHz  
in D2O  
acetic acid sodium as reference

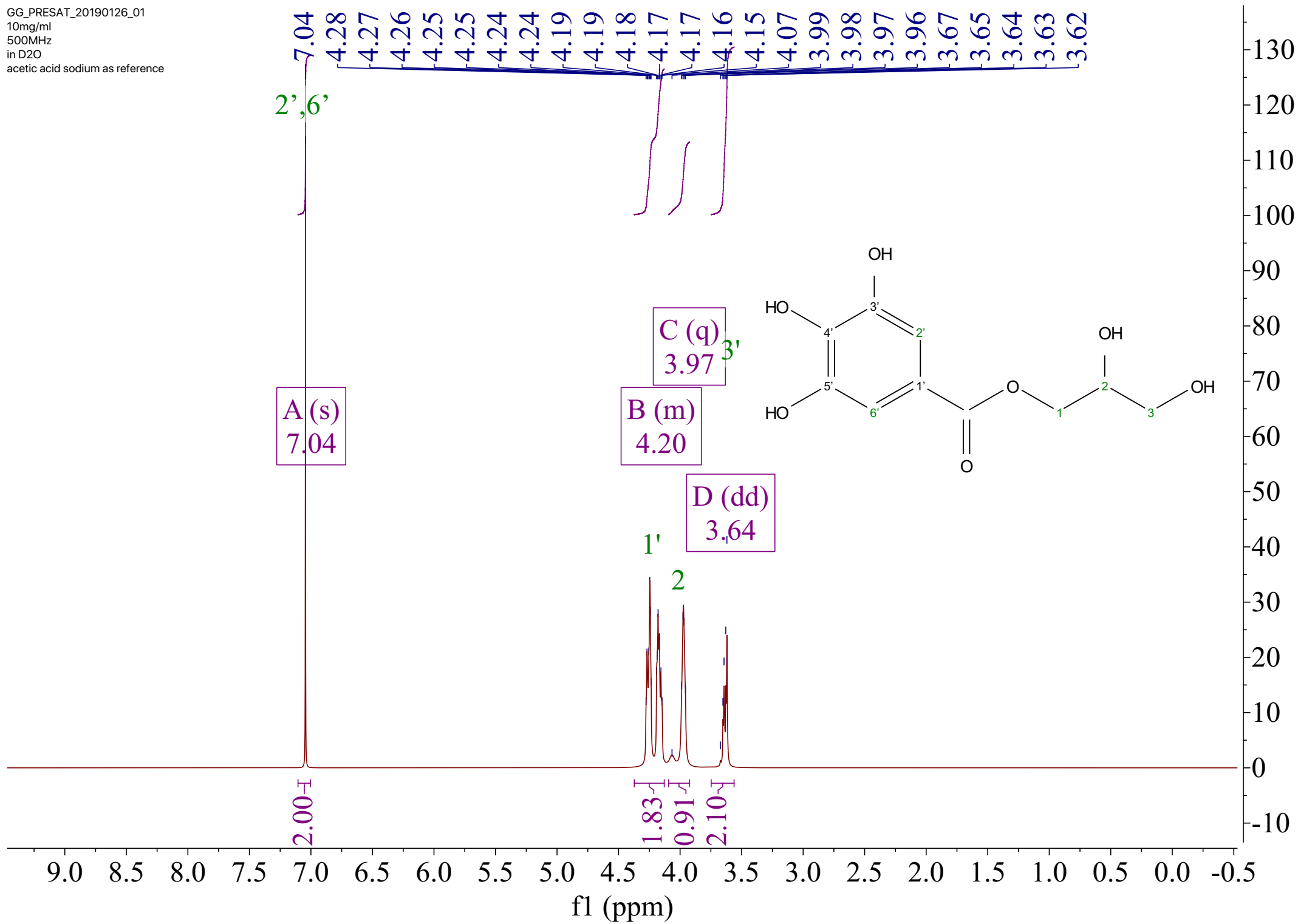

Supplement: Multimedia component 1 [file mmc1.zip › Supplementary materials/NMR/H.pdf]

GG\_gCOSY\_20190127\_01  
10mg/ml  
500MHz  
in D2O  
acetic acid sodium as reference

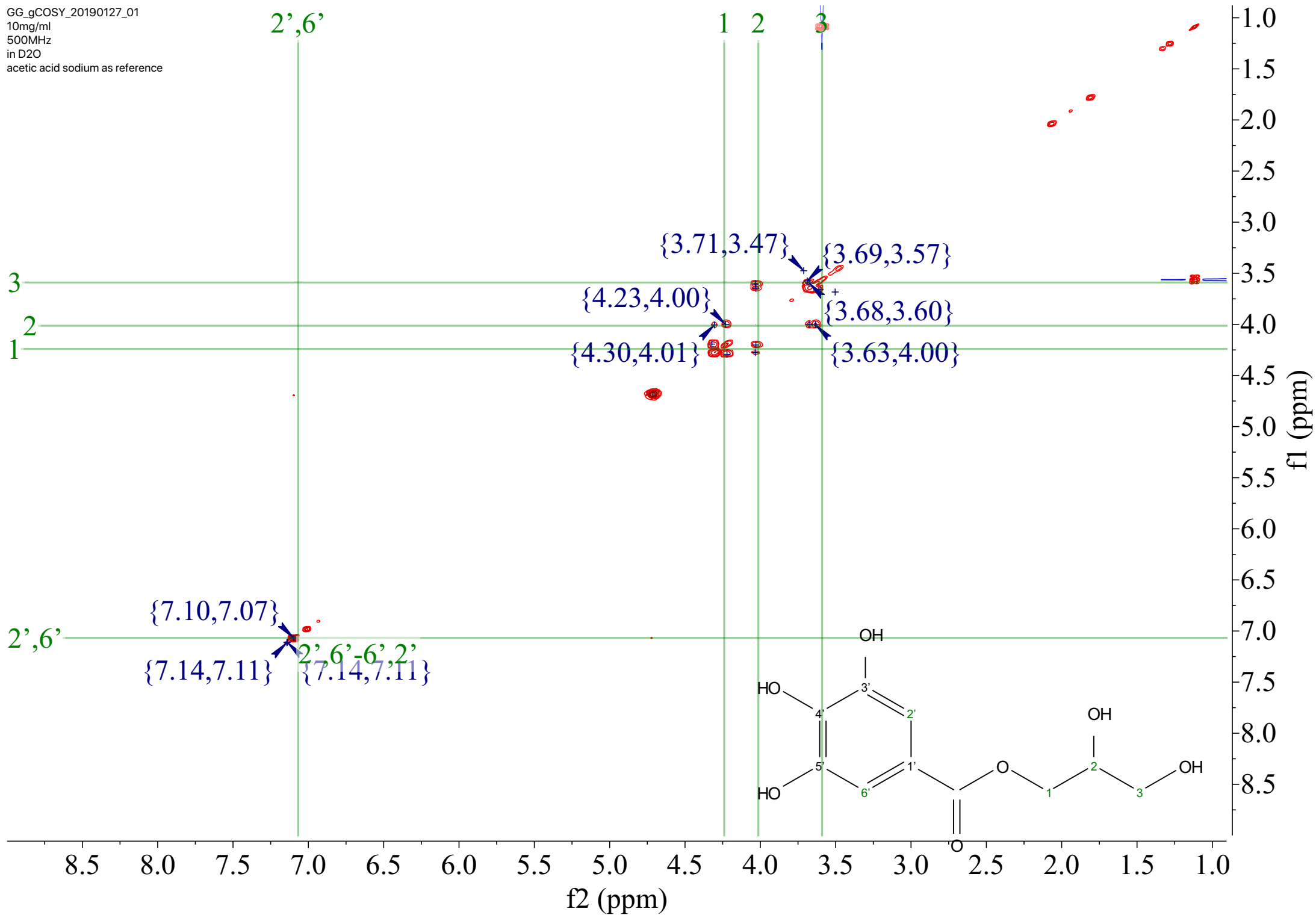

Supplement: Multimedia component 1 [file mmc1.zip › Supplementary materials/NMR/gCOSY.pdf]

GG\_gHMQC\_20190127\_01  
10mg/ml  
500MHz  
in D2O  
acetic acid sodium as reference

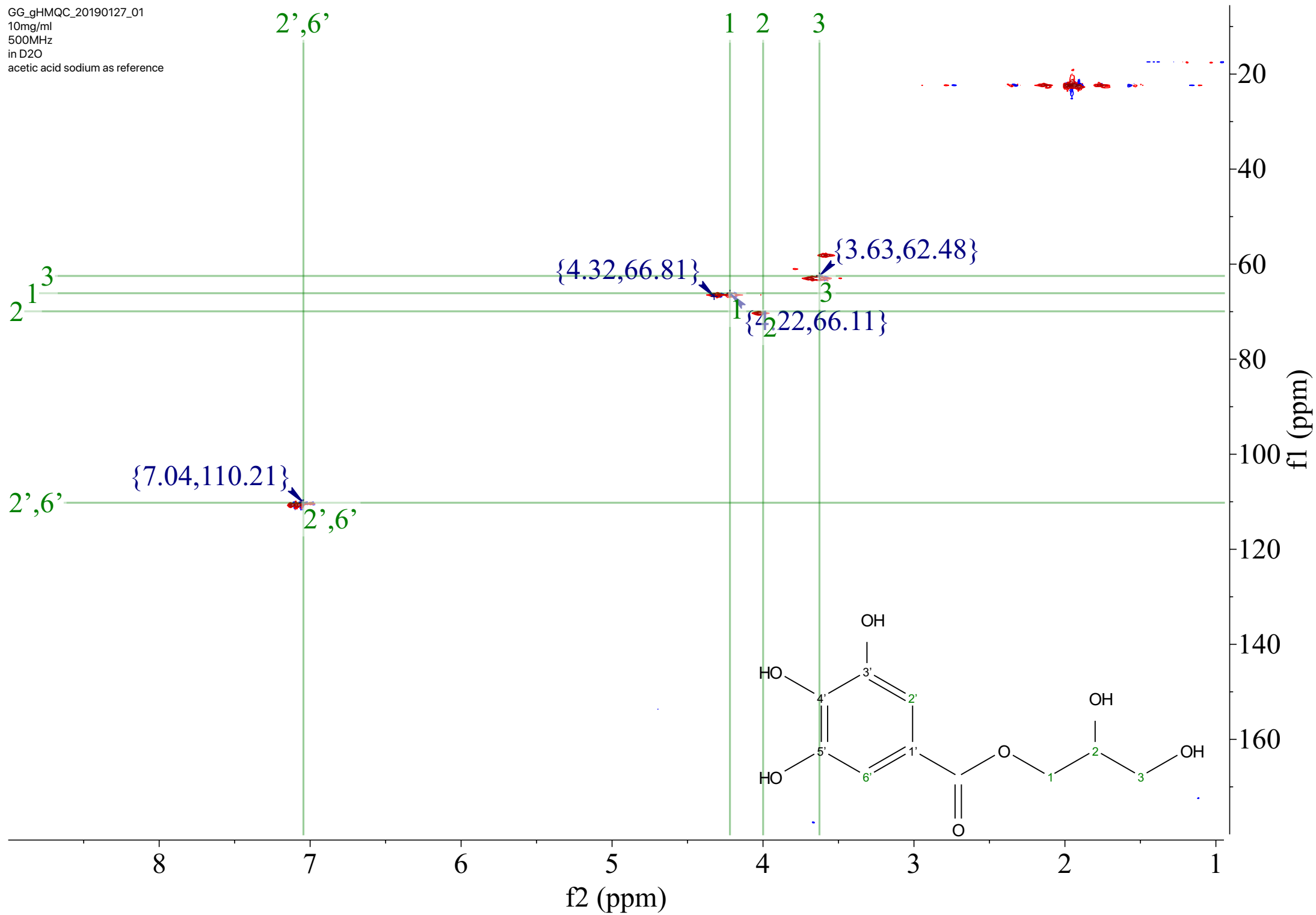

Supplement: Multimedia component 1 [file mmc1.zip › Supplementary materials/NMR/gHMQC.pdf]

GG\_gHSQC\_20190127\_01  
10mg/ml  
500MHz  
in D2O  
acetic acid sodium as reference

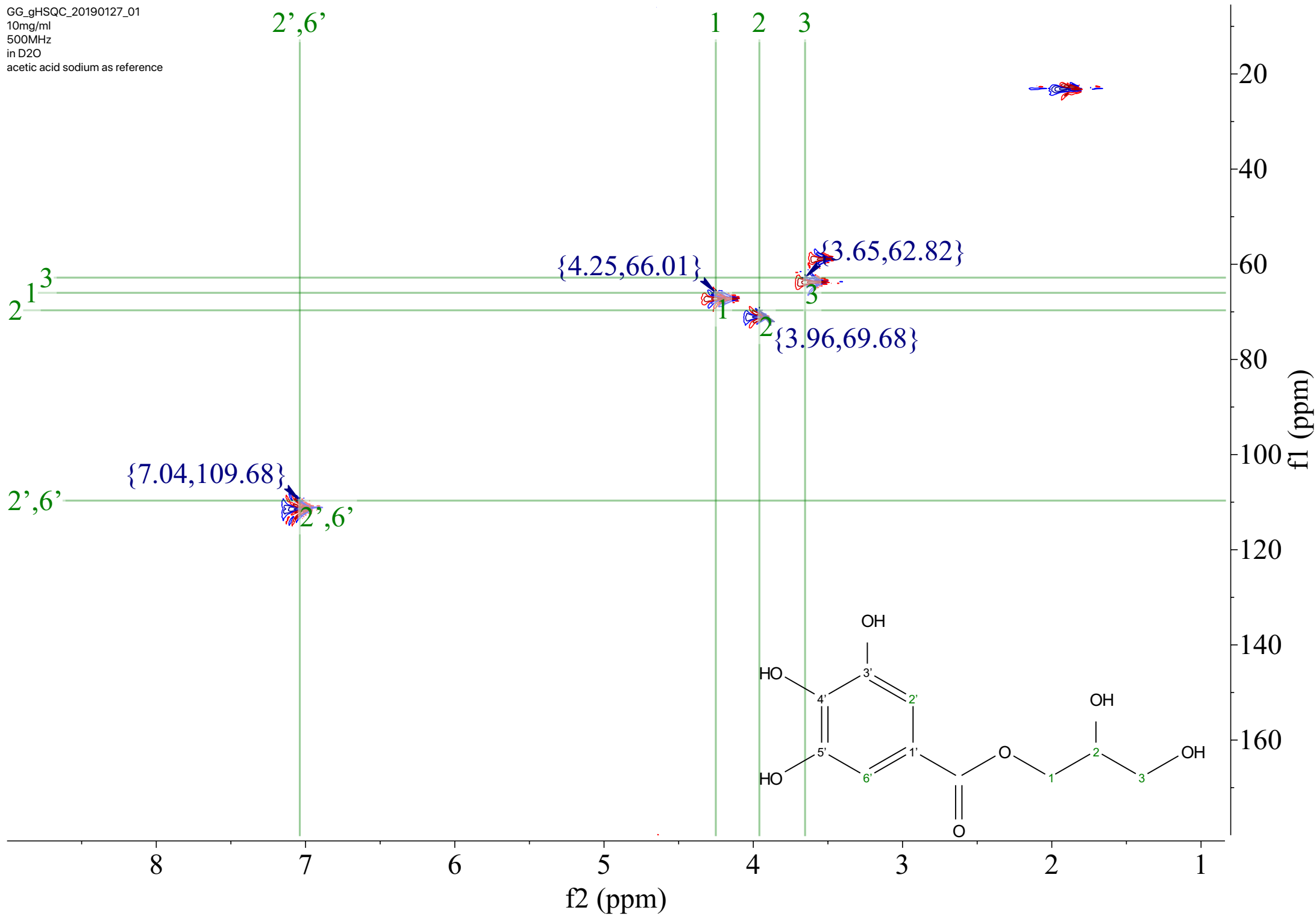

Supplement: Multimedia component 1 [file mmc1.zip › Supplementary materials/NMR/gHSQC.pdf]

GG\_gHMBC\_20190127\_01  
10mg/ml  
500MHz  
in D2O  
acetic acid sodium as reference

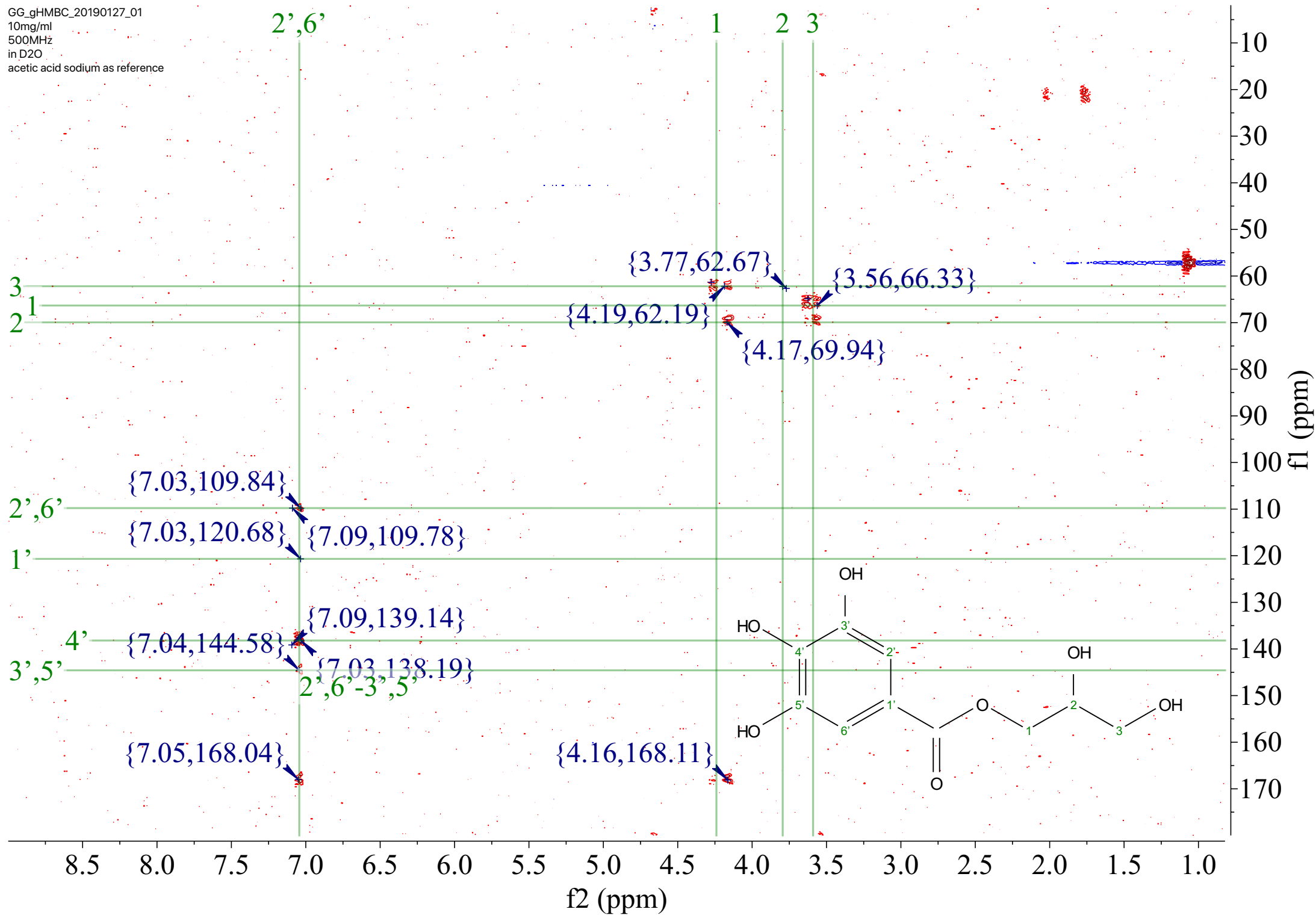

Supplement: Multimedia component 1 [file mmc1.zip › Supplementary materials/NMR/gHMBC.pdf]
